# Consistency verification and interpretation of explainable AI for predicting annual home runs of professional baseball players from sensor data

**DOI:** 10.1038/s41598-025-18403-1

**Published:** 2025-09-25

**Authors:** Shohei Shibata, Yuto Kase, Yusuke Yoshikawa, Kai Ishibe, Takaki Nomoto, Tetsuya Ijiri, Yasuhiro Tahara, Yuta Yamaguchi

**Affiliations:** 1 Global Equipment Product Department, Mizuno Corporation, Suminoe-ku, Osaka, 559-8510 Japan; 2 Global Research & Development Department, Mizuno Corporation, Suminoe-ku, Osaka, 559-8510 Japan; 3 Hokkaido Nippon-Ham Fighters, KitaHiroshima, 061-1116 Hokkaido Japan

**Keywords:** Biomechanics, Bat speed, Bat mass, Random forest, Engineering, Applied physics

## Abstract

This study aimed to verify and interpret a model for predicting the number of home runs per year using sensor data from professional baseball players during batting practice. A machine learning model was constructed using Random Forest from the bat kinematics and bat mass data of 41 professional baseball players collected by a bat-mounted sensor. Partial Dependence analysis and Feature Importance analysis by SHAP (SHapley Additive exPlanations) were used to explain the model’s predictions. The predictive model showed that the bat speed, bat mass, and rotational acceleration are particularly important. The results indicated that a bat speed of 33.3 m/s and rotational acceleration exceeding 157 m/s^2^ exhibited a trend toward a rapid increase in the number of predicted home runs per year. The mass of the bat suggests that an optimum value exists at 0.91 kg. These results suggest that batters who are expected to hit a large number of home runs each year increase the acceleration at the beginning of their swing to produce high bat speed in a short period of time and achieve bat speeds of 33.3 m/s or more with a bat that is somewhat heavier.

## Introduction

 In baseball, the key to victory is to score as many runs as possible. Home runs are expected to contribute greatly to victory, as multiple runs can be scored at once. In addition, home runs covering substantial distances, which are hit in a large arc, are one of the most exciting aspects of baseball and attract spectators. A home run is a longer distance hit compared to a non-home run hit, and the dynamic factors that determine this distance are determined by three parameters: the initial velocity, launch angle, and angular velocity of the batted ball^1,2^. In addition, these parameters are defined by the following four factors^3^: (1) pitching characteristics that can be quantified by the translational and angular velocities of the pitched ball just before impact, (2) impact parameters that represent the positional relationship between the ball and bat at impact, (3) translational and angular velocities of the bat just before ball impact, and (4) dynamic characteristics of the bat, which are expressed by mass, moment of inertia, material characteristics, etc. Among these, data related to the mass of the bat used for measurement by individual players and the swing characteristics of individual players can now be easily measured in large quantities at the field level, as advances in sensor technology have made sensors smaller and less expensive. Wireless inertial measurement unit (IMU) sensors that can be attached to the knob of a bat have been developed, and several manufacturers have commercialized IMUs that measure the speed and trajectory (swing angle) of the bat^4^. They can record the length and mass of the bat at the time of measurement. However, it is not clear how these parameters relate to the actual hitting performance in the game, such as the number of home runs per year and the contribution of each parameter. By clarifying the relationship between biomechanical parameters in the practice environment and the number of home runs per year, the parameters that players should focus on will become clear. This information will be very useful as players aim to strengthen their skills. For coaches, the advanced prediction of the number of home runs per year can lead to rational tactical construction and coaching. If an advance prediction model of the number of home runs per year could be created, it would be possible to suggest effective training programs, such as encouraging strength training for players predicted to hit more home runs and those who are predicted to hit fewer home runs to focus more on ball contact and spend more time on base running and defense. In particular, it could be used in terms of early assessment of player development policies for young players and newcomers with no past performance.

Traditionally, sports science research has been dominated by studies on sports biomechanics and sports physiology; however, recently, efforts have also been made to incorporate machine learning, which has a high predictive performance for large amounts of data. Tree ensemble methods, such as random forests (RF), have been successfully used in biomechanical research owing to their robustness^5,6^. Therefore, it would be useful to use machine learning methods to predict the number of home runs per year based on biomechanical parameters in a practice environment.

Nevertheless, such tree ensemble algorithms can be more difficult in interpretation, despite their potentially satisfactory prediction performance. This drawback can be mitigated using model interpretation techniques, such as explainable artificial intelligence (XAI), thus providing insights into algorithm predictions^7^. However, it has not been applied to kinematic data pertaining to the hitting motion in baseball. Thus, this study aimed to verify a model for predicting the annual number of home runs using sensor data from professional baseball players and interpret it by applying XAI.

In this study, a hypothesis for the research purpose is designed based on the findings of several previous studies. In a study on swing analysis using a high-speed camera for batting practice by college players^3^, a multiple regression equation was presented in which the distance of the batted ball was the objective variable and two variables, bat head speed and the angle between the velocity vector of the bat head and the horizontal line, were the explanatory variables. The contribution ratio was 52%, and the standardized partial regression coefficients were 0.64 (bat head speed) and 0.31 (angle of the bat swing), respectively. In addition, to increase the velocity of the batted ball, it is necessary to increase not only the bat speed but also the equivalent mass at the hitting position^8^. The equivalent mass at the hit position was large because of the larger mass and moment of inertia of the bat. Thus, it was hypothesized that the home-run prediction model based on data acquired by the IMU sensors made significant contributions to the bat speed and bat mass.

## Results

### Comparison of measured and predicted values

The coefficient of determination ($\:{R}^{2})$ and root mean square error (RMSE) values of the trained RF, multiple regression analysis, and baseline models (a mean value model that predicts the mean value of the objective variable in the training data) were calculated (Table 1). $\:{R}^{2}$ of the training data in RF is 0.65 and $\:{R}^{2}$ of the test data in RF is 0.56$\:\pm\:0.08$. $\:{R}^{2}$ of the trained RF was the highest, and the RMSE was the lowest (RMSE = 5.30$\:\pm\:0.51$).

**Table 1:** $\:{R}^{2}$ And RMSE of the trained RF, gradient boosting (GB), multiple regression analysis, And baseline models.

Model	$\:{R}^{2}$	RMSE
RF	0.56 ± 0.08	5.30 ± 0.51
GB	0.49 ± 0.04	5.68 ± 0.26
Multiple Linear Regression	0.52 ± 0.05	5.54 ± 0.30
Base-line model	-	8.27

## Interpretation by XAI

**Fig. 1:**
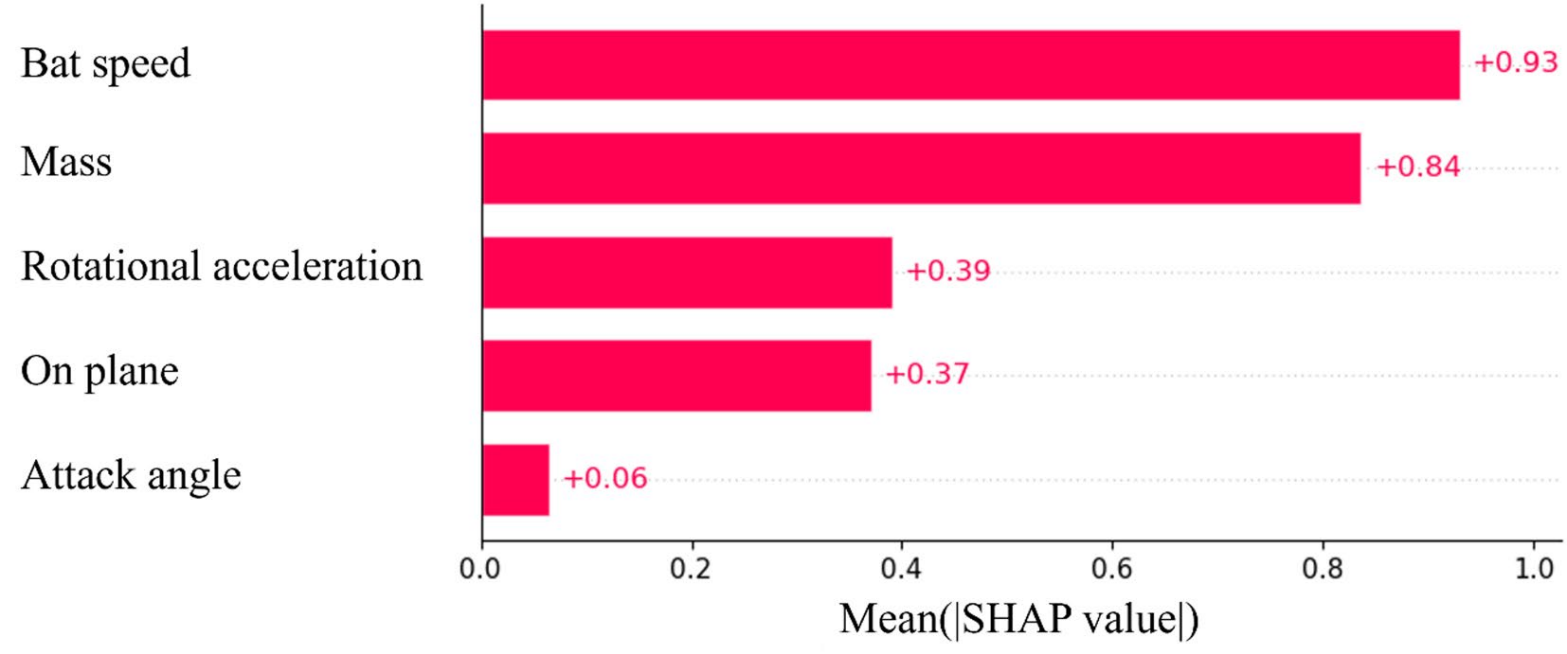
Mean importance of SHAP for the model. The horizontal axis indicates the mean importance of SHAP, and the vertical axis indicates each explanatory variable.

**Fig. 2:**
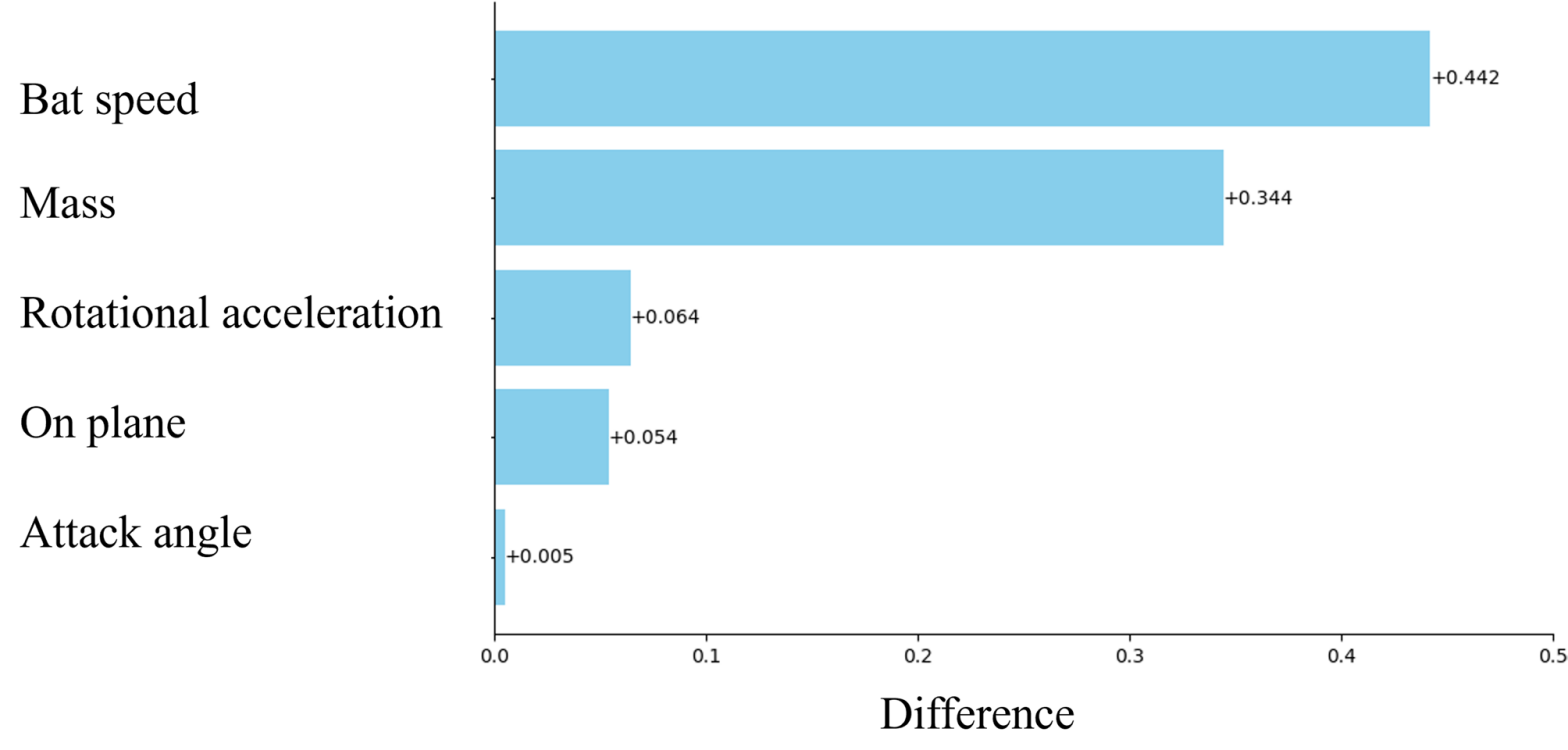
Permutation Feature Importance. The horizontal axis indicates difference in prediction accuracy due to Permutation Feature Importance, and the vertical axis indicates each explanatory variable.

**Fig. 3:**
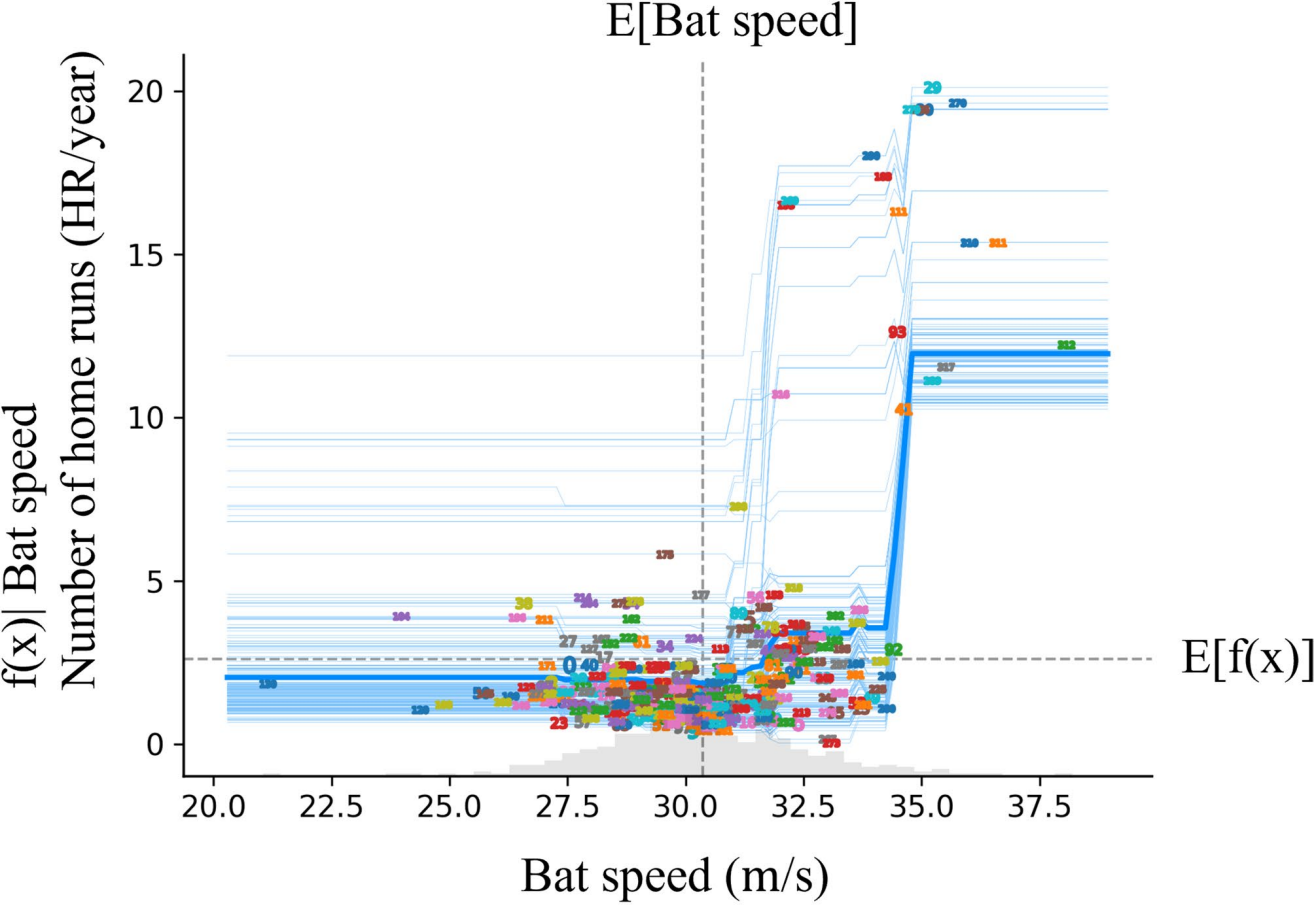
Result of the PD and ICE for bat speed. The horizontal axis represents the bat speed, and the vertical axis represents the predicted number of home runs per year. Dashed lines in the graph indicate the expected values. The thick blue line shows the mean relationship between the explanatory variables and the predicted values ​​when the explanatory variables are replaced, and the blue line shows the relationship between the explanatory variables for each instance and the predicted values ​​when the explanatory variables are replaced. The numbers on blue lines mean each instance.

**Fig. 4:**
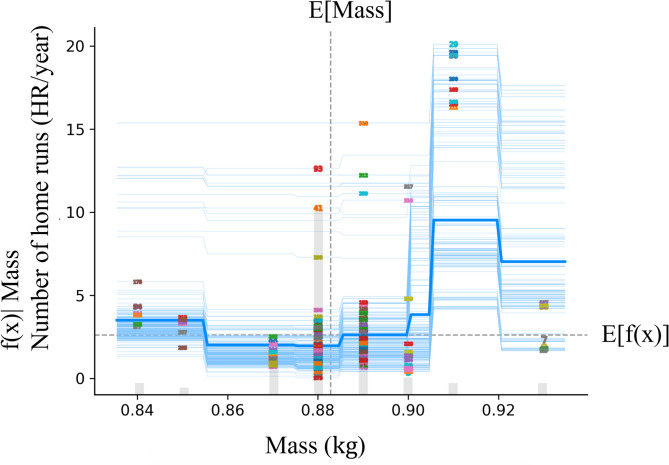
Result of the PD and ICE for the mass of the bat. The horizontal axis shows the mass of the bat and the vertical axis shows the predicted number of home runs per year. Dashed lines in the graph indicate the expected values. The thick blue line shows the mean relationship between the explanatory variables and the predicted values ​​when the explanatory variables are replaced, and the blue line shows the relationship between the explanatory variables for each instance and the predicted values ​​when the explanatory variables are replaced. The numbers on blue lines mean each instance.

**Fig. 5:**
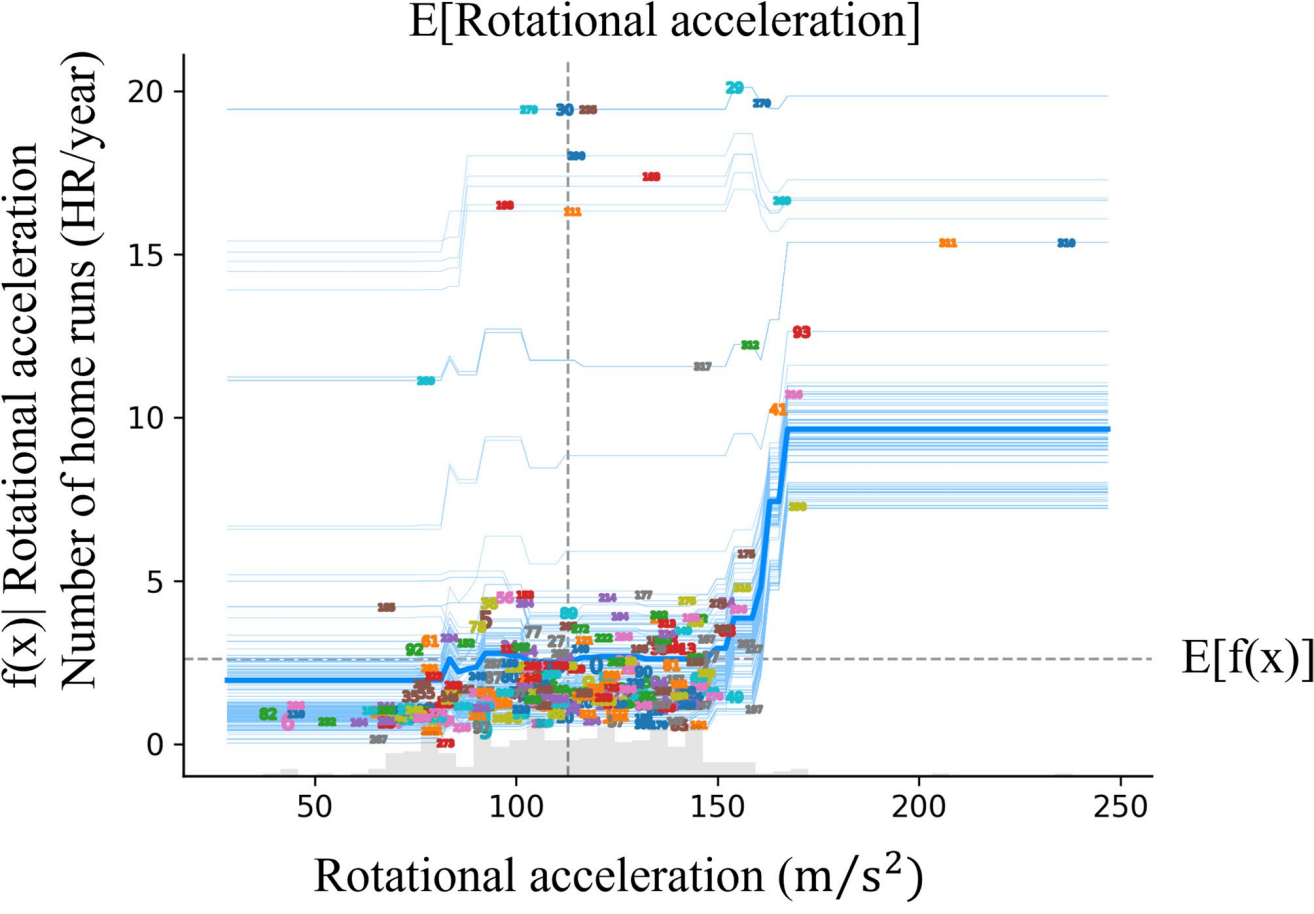
Result of the PD and ICE for rotational acceleration. The horizontal axis represents rotational acceleration, and the vertical axis represents the predicted number of home runs per year. Dashed lines in the graph indicate the expected values. The thick blue line shows the mean relationship between the explanatory variables and the predicted values ​​when the explanatory variables are replaced, and the blue line shows the relationship between the explanatory variables for each instance and the predicted values ​​when the explanatory variables are replaced. The numbers on blue lines mean each instance.

**Fig. 6:**
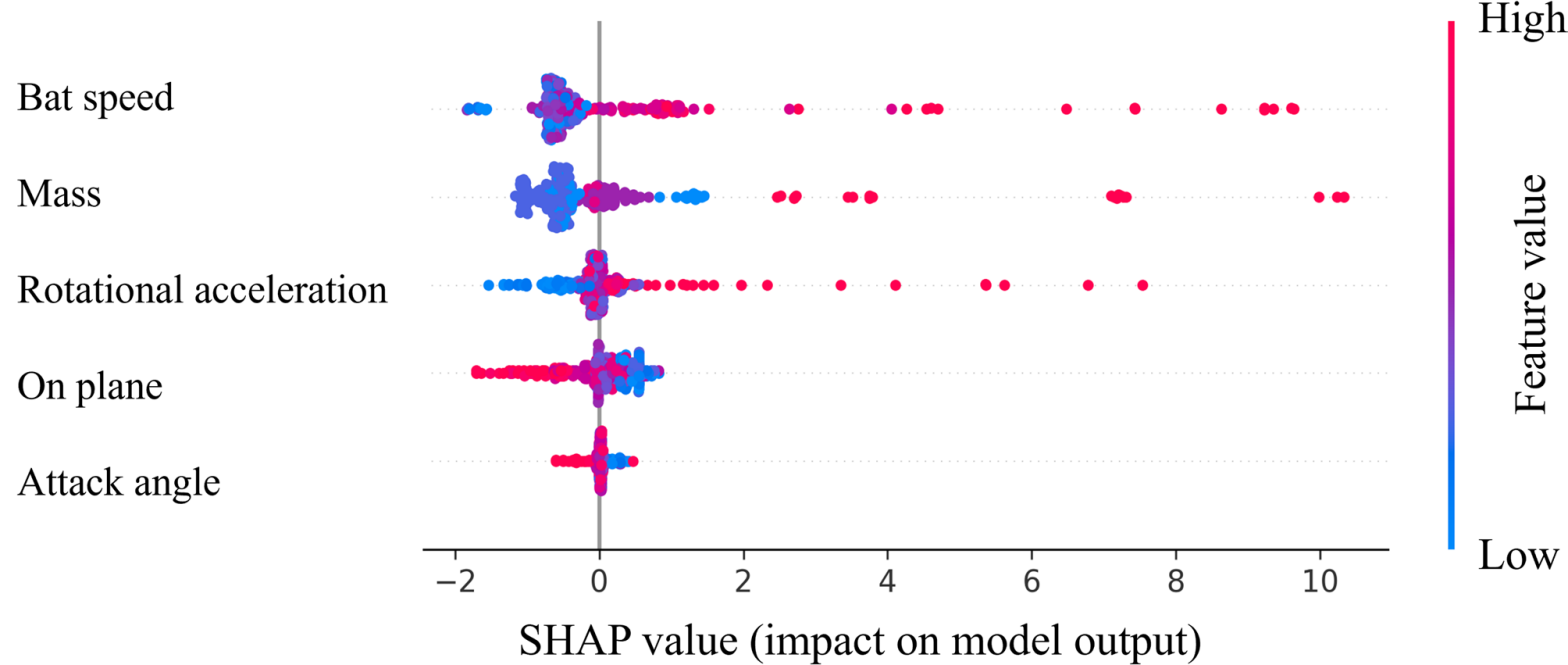
SHAP beeswarm plots. The vertical axis represents each explanatory variable and the horizontal axis represents the SHAP value, which is an index showing the effect of features on the prediction model. Each point represents an individual dataset.

The mean importance of SHAP for the model is shown in Fig. 1. The feature of bat speed appears to be the most important parameter for prediction. The mass of the bat was the second most important factor, and the rotational acceleration was the third most important. From this point, the focus is on these three parameters. Similar to the SHAP, Permutation Feature Importance showed similar results (Fig. 2). Figures 3, 4 and 5 show the mean relationship between the explanatory variables and the predicted values ​​when replacing the explanatory variables (partial dependence: PD) and the relationship between the explanatory variables for each instance and the predicted values ​​when replacing the explanatory variables (Individual Conditional Expectation: ICE). PD showed that the predicted number of home runs per year increased rapidly at bat speeds above 33.3 m/s (Fig. 3). The PD results for bat mass (Fig. 4) showed that the optimum value is 0.91 kg. The PD results for the rotational acceleration (Fig. 5) show that a rotational acceleration of over 157 m/s^2^ leads to a sharp increase in the predicted number of home runs per year. Figure 6 shows the results of the SHapley Additive exPlanations (SHAP) value, which is an index indicating the effect of features on the prediction model.

## Discussion

The purpose of this study is to verify a model for predicting the number of home runs per year using sensor data of professional baseball players and interpret the model by applying XAI to batting practice, which is a practice environment for baseball. Comparing the measured and predicted number of home runs per year in the training and test data obtained from the trained RF, the $\:{R}^{2}$ of the training data was 0.65 and that of the test data was 0.56$\:\pm\:0.08$, indicating that the model was a good fit despite some overfitting. The $\:{R}^{2}$ and RMSE of the trained RF, multiple regression analysis, and baseline models were calculated. The $\:{R}^{2}$ of the trained RF was the highest, and the RMSE was the lowest (RMSE = 5.30$\:\pm\:0.51$), indicating that it can predict the number of home runs per year more accurately than conventional methods. There were two factors that made it possible to predict the game result with high accuracy from the practice data: First, the data from the practice environment were the flying ball hitting condition by batting practice pitchers. Koenig et al. (2004)^9^ compared the batting motion of various bats under tee-hitting conditions with that of hitting a pitched ball. The results showed that most participants had less bat speed when hitting a pitching ball than when tee-hitting condition, and the degree of speed loss differed between participants. If the practice data were obtained under the tee-hitting condition, the swing would be overestimated and it would be difficult to reproduce the model with high accuracy. The second is the number of data. In this study, all swing observations were treated as independent. On the other hand, when the mean of ten swings per player was used in the analysis, the accuracy of the model was greatly reduced (The $\:{R}^{2}$ of the trained RF was 0.22**)**. The results suggest that all observed swings were considered independent, and that a significant increase in the amount of data contributed to the improved accuracy. In the model used for predicting the number of home runs per year, bat speed was the most important feature, followed by bat mass and rotational acceleration. This result supports the hypothesis that bat speed and mass are significant contributors to home-run prediction using the model.

The PD results showed a sharp increase in the number of predicted home runs per year starting at bat speeds above 33.3 m/s. To validate this result, we estimated the conditions necessary to hit a home run at 100 m, according to the laws of physics. Once a ball becomes ballistic, that is, once it is released by its launching mechanism, only gravitational and aerodynamic forces act on the ball^10^. The vector equation of motion that models the center-of-mass trajectory is

1 $$\:m\varvec{g}+\varvec{L}+\varvec{D}+\varvec{Y}=m\ddot{\varvec{x}}$$

where *m* is the mass of the ball (0.14 kg), $\:\varvec{g}$ is the gravitational acceleration vector, $\:\varvec{L}$ is the lift force vector, $\:\varvec{D}$ is the drag force vector, $\:\varvec{Y}$ is the side force vector, and $\:\varvec{x}$ is the position vector of the center of mass of the ball. ***L*** was assumed to be perpendicular to both the angular and translational velocity vectors and was characterized in terms of a dimensionless lift coefficient $\:{C}_{L}$:

2 $$\:\varvec{L}=\frac{\rho\:{C}_{L}A{\parallel\dot{\varvec{x}}\parallel}^{2}}{2}\frac{\varvec{\omega\:}\times\:\dot{\varvec{x}}}{\parallel\varvec{\omega\:}\times\:\dot{\varvec{x}}\parallel}$$

where $\:\rho\:$ is the mass density of air, $\:A\:$is the cross-sectional area of the ball, $\:\varvec{\omega\:}$ is the angular velocity vector of the ball. Similarly, drag $\:\varvec{D},$ is characterized by the drag coefficient $\:{C}_{D}$, and acts in the direction opposite to that of the translational velocity vector:

3 $$\:\varvec{D}=-\frac{\rho\:{C}_{D}A\parallel\dot{\varvec{x}}\parallel\dot{\varvec{x}}}{2}$$

The side force $\:\varvec{Y}$, is characterized by a third dimensionless side force coefficient,$\:\:{C}_{Y}$:

4 $$\:\varvec{Y}=\frac{\rho\:{C}_{Y}A{\parallel\dot{\varvec{x}}\parallel}^{2}}{2}\frac{\varvec{L}\times\:\varvec{D}}{\parallel\varvec{L}\times\:\varvec{D}\parallel}$$

The cross-product term in Eq. (4) ensures that $\:\varvec{Y}$ is perpendicular to $\:\varvec{L}$ and $\:\varvec{D}$. The lift, drag and side-force coefficients were calculated as a function of the spin parameter $\:\frac{r\parallel\varvec{\omega\:}\parallel}{\parallel\dot{\varvec{x}}\parallel}$ depending on the azimuth angle of the spin axis. Using Eqs. (1)–(4), the ball position during flight was calculated by integration.

**Fig. 7:**
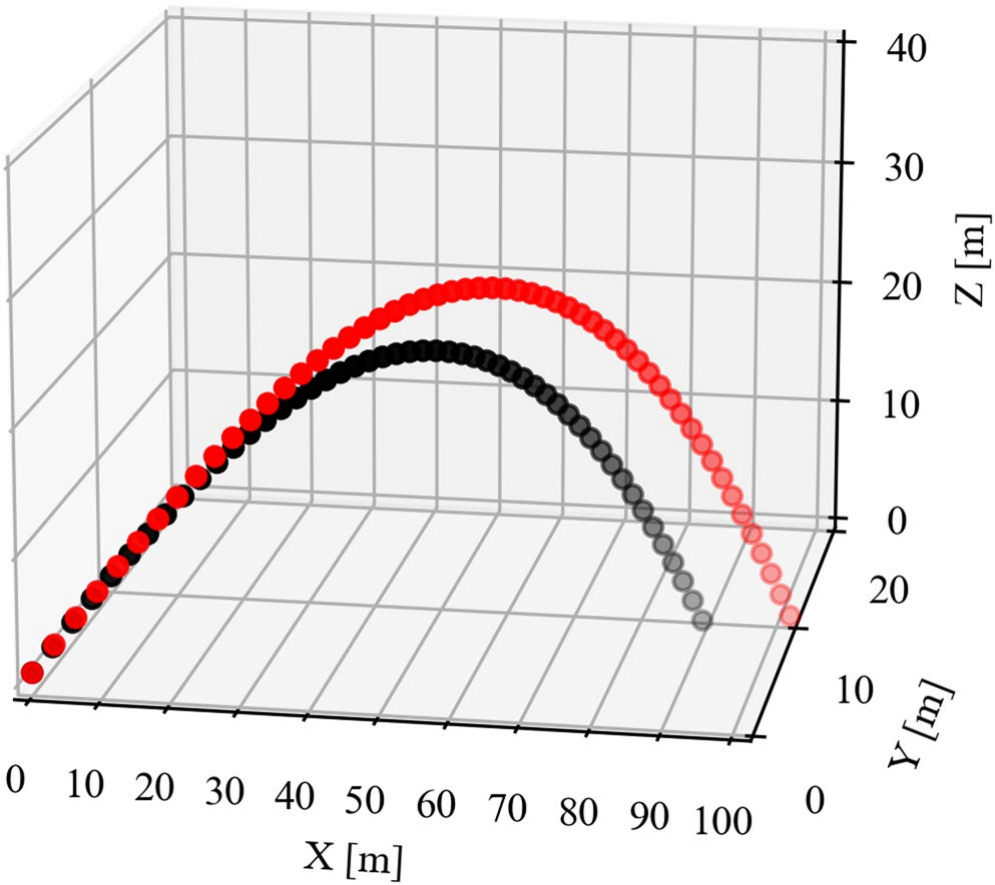
Ballistic simulation of different batted ball velocity. The x-axis represents the direction of travel of the ball, the y-axis represents the left-right direction, and the z-axis represents the ball height. The red circle is the result of a homerun with a distance of 100 m at a batted ball velocity of 40.8 m/s ($\:{C}_{L}=0.21,\:{C}_{D}=0.40,\:{C}_{Y}=-0.06$). The black circle is the result of a distance of 89 m at a batted ball velocity of 37.5 m/s ($\:{C}_{L}=0.23,\:{C}_{D}=0.40,\:{C}_{Y}=-0.05$). All other conditions were identical.

Here, as an initial condition for estimating the flight distance, the kinematic data of a mean flight distance of 88 m hit by a university baseball player to left field were used^11^; the initial launch angle was 33.2°, and the initial angular velocity was 225 rad/s. The spin axis of the batted ball had an elevation angle of 13.3° and azimuth angle of 15.4°. The initial height of the batted ball was 1.0 m. Under these conditions, the calculations began with a batted ball speed of 37.5 m/s, and by increasing it by 0.28 m/s each time, the batted ball speed that resulted in a distance of over 100 m for the first time was calculated. As a result, it was calculated that a batted ball velocity of 40.8 m/s ($\:{C}_{L}=0.21,\:{C}_{D}=0.40,\:{C}_{Y}=-0.06$) or more would be required to hit a home run with a distance of 100 m (Fig. 7). The lift, drag, and side force coefficients refer to values from previous studies^12^. In addition, a previous study^13^ investigated the relationship between batted ball speed and bat head speed, and the regression equation (y = 0.865x + 6.425; y: batted ball speed, x: bat head speed) shows that to achieve a batted ball speed of 40.8 m/s, a bat head speed of about 39.7 m/s is required. The IMU (BLAST BASEBALL) fixed to the bat in this study calculates the speed at a position 0.15 m from the head (the distal end of the bat) toward the grip (the proximal end)^4^. Therefore, assuming that 0.28 m/s occurs for every 0.01 m, BLAST BASEBALL calculates a value that is approximately 4.2 m/s less than the bat head speed. As a result, if the bat head speed is 39.7 m/s, the BLAST bat speed required to hit a home run at a distance of 100 m is expected to be approximately 35.5 m/s. This result is similar to the finding in this study that a bat speed of over 33.3 m/s leads to a sharp increase in the predicted number of home runs per year (the difference is approximately 6.1%), and indicates that a reasonable relationship can be derived from the results obtained using machine learning.

The PD results for the bat mass showed that the optimal value is 0.91 kg. A previous study using bats with large and small masses^14^ reported that the bat speed decreased with a large-mass bat. By contrast, long-distance hitters who hit many home runs per year may expect to improve their flying distance by increasing the apparent mass of the bat at the ball impact point, which is the mass of the bat at the ball impact position, even if the increase in mass leads to a decrease in operability and bat speed immediately before impact. The optimal value for these factors is 0.91 kg.

The PD results for rotational acceleration showed that a rotational acceleration of over 157 m/s^2^ leads to a sharp increase in the predicted number of home runs per year. Koike and Mimura^15^ performed a dynamic analysis of the bat speed movement and showed that the contribution of the joint torque term was largest in the first half of the swing. In other words, rotational acceleration in the first half of the movement may be related to the output of the joint torque generated by muscle tension, and a long-distance hitter is expected to have a large rotational acceleration value. In baseball batting, it is necessary to predict the type of ball and its trajectory, instantly decide whether to swing, and swing accurately to the desired location within as short a swing time as possible. Even if the two batters have the same bat speed at impact, if the bat accelerates faster at the beginning of the swing, the batter will be able to achieve impact with the ball in a shorter time, allowing more time to predict the ball trajectory and make swing decisions, which is an advantage in batting motion. For these reasons, the importance of rotational acceleration is considered to be high in the home-run number prediction model.

## Limitation

In this study, data from different players in different years were used as training and test data, which showed a certain generalization performance. However, the objective variable in this study is the number of home runs per year, an indicator that includes not only a player’s kinematic swing characteristics but also the effects of other factors such as ball-and-bat contact skills and ballpark size. In addition, home-run totals are heavily influenced by both the number of at-bats and the quality of opposing pitchers. For example, skill differences in opposing pitchers in different leagues or bias in the types of batters (e.g. power vs. contact hitters) sampled and trained could lead to overfitting. Therefore, it is necessary to verify whether a similar generalization performance is demonstrated for data on players from different leagues, age groups, competitive levels and non-Japanese professional baseball players. Also, future study would need to conduct longitudinal studies and incorporate game-contextual variables (pitch type, opponent, game situation).

## Methods

### Experimental design

Forty-one male Japanese professional baseball players (age: 26 ± 4 years; weight: 85 ± 10 kg; height: 1.81 ± 0.06 m) participated in the experiment. All the participants provided informed consent before participating in the study. The experimental procedure was approved by the Ethics Committee of Mizuno Corporation (Approval ID: 20241203-312). The players gave consent for the publication of anonymized results. All methods were performed in accordance with the relevant guidelines and regulations. For practical data collection in the field, rather than in a laboratory environment, data were collected using batting practices thrown by batting practice pitchers during professional baseball spring camp practice. Batting practice pitchers threw a four-seam fastball. Thirty-one professional baseball players were assessed in the spring camp of 2023, and 10 players were assessed in the spring camp of 2024. After warming up according to their normal routine, each batter performed a batting motion using a bat fitted with an IMU. We analyzed the swing data for each player over ten trials.

## Recording movement

An IMU (BLAST BASEBALL, BLAST Motion, USA) was attached to the bat knob. The version of the application software was 5.14 in Japan. The participants used wooden bats (mass:0.88$\:\pm\:0.02$ kg length: 0.85$\:\pm\:0.01$ m) that were normally used by players. The IMU was fixed to the bat using a special attachment provided by the manufacturer. The bat mass information was input into a dedicated application software downloaded to the tablet, and the bat swing was measured using the software. The accuracy of the bat speed and attack angle was confirmed through a comparison with the results obtained using an optical motion capture system from a previous study^4^. A strong correlation existed between the bat speed obtained using BLAST BASEBALL and that obtained using the motion capture system (*r* = 0.91, ICC = 0.67). A moderate correlation existed between the attack angle obtained by BLAST BASEBALL and that obtained using the motion capture system (*r* = 0.59, ICC = 0.45). 40% of the participants in the previous study were professional baseball players, an elite group, and that the data included bat speeds in the high velocity range of nearly 35 m/s. Based on this result, it was concluded that BLAST BASEBALL is applicable to the data in this study of professional baseball players.

## Pre-processing

Ball impact is determined when the sensor “trigger” measured on the accelerometer and gyroscope exceeds specific thresholds for impact. The “impact” index is the sample before (2 ms) the sensor registers this shock. Five features were extracted from the IMU data.

1. The bat speed in BLAST BASEBALL was calculated from the displacement of the coordinates of a position 0.15 m away from the bat head in the direction of the knob at impact.

In addition, speed was calculated as the magnitude of the velocity perpendicular to the direction of the bat. 2. Mass of bats used by each player. 3. The attack angle was calculated as the angle between the velocity vector 0.15 m away from the bat head in the direction of the knob and the horizontal plane. 4. The on-plane efficiency is the ratio between the angular velocity in the direction of contact and the total angular velocity of the bat (excluding the bat roll), which is expressed as a percentage. 5. Rotational acceleration is the acceleration of the bat measured from the point at which the knob first begins to accelerate downwards over a predetermined window. In other words, rotational acceleration is acceleration, not angular acceleration. This metric measures how quickly a player initiates rotation of the swing. This representation (rotational acceleration) is the notation on the IMU application (BLAST BASEBALL) used in this study. All five features were used simultaneously as input in the RF model.

Of these five features, the three mechanical factors that directly affect flight distance are bat speed, which has a significant impact on batted ball speed; bat mass, which is the inertial characteristics of the bat; and attack angle, which is related to the launch angle. On the other hand, to increase the number of home runs per year, which is the objective variable, it was considered that features reflecting not only the mechanical factor that increases flight distance but also the skill to handle all types of pitches multiple times would be necessary. On-plane efficiency, which represents the percentage of the trajectory in motion on a single plane during the swing, is assumed to be related to the rate of contact between the ball and the bat at all points during the swing and its stability. In addition, a larger value of rotational acceleration may be advantageous in dealing with all types of pitches, as the same bat speed may result in a shorter time to impact. Based on the above, on-plane efficiency and rotational acceleration were added to the features as they are expected to contribute to improving the prediction performance of the machine learning model.

The mean bat speed was calculated for each player, and data that deviated from the mean by more than 2.78 m/s were excluded as outliers. The number of home runs in the first team’s seasonal performance was used as the objective variable. The annual results for each player were extracted from the Nippon Professional Baseball Organization website^16^. Since RF is a method that does not require standardization of features, the value of the number of home runs per year was used as is. The average number of home runs was 3.0 $\:\pm\:$ 6.1.

### Machine learning with random forest

RF were used to regress the number of home runs per year. This algorithm combines many decision trees grown in random subsets selected by bootstrap aggregation from the feature space^7,17^. Human swing motion is expected to include complex interactions between features (such as the relationship between bat speed and mass of bat) that are difficult to capture with linear regression, and a nonlinear analysis method such as RF is expected to be appropriate. In addition, the RF has been shown to provide a higher accuracy than other existing Machine Learning with a faster computation speed^5^. Also, RF have been successfully used in biomechanical research because of their robustness^5,6^. From these reasons, the nonlinear method RF was applied in this study. Model results validation was checked by splitting the data into training (90%) and testing (10%) datasets. A total of 295 swings from 31 players measured at the spring training camp in 2023 were used as the training data, and 30 swings from another 10 players measured at the spring training camp in 2024 were used as the test data. In the test data, three trials were randomly selected for each player, and the model was constructed. This was done a total of 10 times, and the mean and standard deviation of $\:{R}^{2}$ and RMSE of the model was calculated. Hyperparameter tuning was performed, and there were ten decision trees and the maximum depth was five. To compare the performances of the models, we performed a gradient boosting (GB) and a multiple regression analysis using the same dataset. In GB, hyperparameter tuning was performed, and there were fifty decision trees and the maximum depth was five. In multiple regression analysis, explanatory variables were standardized. Similarly, we calculated the RMSE of the mean-value model that predicted the mean value of the objective variable in the training data as a baseline model. All models were built in Python 3.8 with scikit-learn 1.3.0.

### Interpretation by XAI

To interpret the predictions of the adopted RF model, we performed the following three analyses^18^. 1.PD: PD is a method for visualizing the mean predicted value of each instance by varying only the level of a certain feature while fixing the other features. 2. ICE: Unlike PD, which looks at the mean relationship between features and objective variables, ICE is a method for checking the relationship between features and predicted values ​​by varying the level of a certain feature for each instance while fixing the other features^19^. 3.SHAP: SHAP is an application of the Shapley Value, an evaluation method in cooperative game theory for fairly distributing the gains obtained through cooperation among multiple players. SHAP replaces the players in the Shapley Value of cooperative game theory with features and decomposes the difference between the “prediction value for a certain instance” and the “mean prediction value” into the contribution of each feature. The mean importance of each feature was visualized using SHAP. In addition, SHAP beeswarm plots are more complex and information-rich displays of SHAP values that reveal not only the relative importance of features but also their actual relationships with the predicted outcome^20^. Thus, SHAP beeswarm plots were created. As with SHAP, the importance of features by Permutation Feature Importance^18^ was calculated to assess the robustness of the results.

## Data Availability

The anonymized datasets generated and/or analyzed in the current study are available from the corresponding author upon reasonable request.
